# Regional brain gray and white matter changes in perinatally HIV-infected adolescents^☆^

**DOI:** 10.1016/j.nicl.2013.10.012

**Published:** 2013-10-29

**Authors:** Manoj K. Sarma, Rajakumar Nagarajan, Margaret A. Keller, Rajesh Kumar, Karin Nielsen-Saines, David E. Michalik, Jaime Deville, Joseph A. Church, M. Albert Thomas

**Affiliations:** a Department of Radiological Sciences, David Geffen School of Medicine, University of California, Los Angeles, CA 90095, USA; b Los Angeles Biomedical Research Institute at Harbor-UCLA Medical Center, Department of Pediatrics, Torrance, CA 90502, USA; c Department of Anesthesiology, David Geffen School of Medicine, University of California, Los Angeles, CA 90095, USA; d Department of Pediatrics, David Geffen School of Medicine, University of California Los Angeles, CA 90095, USA; e Department of Pediatrics, Miller Children's Hospital of Long Beach, Long Beach, CA 90806, USA; f Department of Pediatrics, Children's Hospital Los Angeles, Los Angeles, CA 90027, USA

**Keywords:** ART, antiretroviral therapy, MRI, magnetic resonance imaging, VBM, voxel based morphometry, GM, gray matter, WM, white matter, SPM, statistical parametric mapping, CSF, cerebrospinal fluid, WMV, white matter volume, GMV, gray matter volume, HIV, Antiretroviral therapy, Voxel based morphometry, Statistical parametric mapping, Gray matter, White matter

## Abstract

Despite the success of antiretroviral therapy (ART), perinatally infected HIV remains a major health problem worldwide. Although advance neuroimaging studies have investigated structural brain changes in HIV-infected adults, regional gray matter (GM) and white matter (WM) volume changes have not been reported in perinatally HIV-infected adolescents and young adults. In this cross-sectional study, we investigated regional GM and WM changes in 16 HIV-infected youths receiving ART (age 17.0 ± 2.9 years) compared with age-matched 14 healthy controls (age 16.3 ± 2.3 years) using magnetic resonance imaging (MRI)-based high-resolution T1-weighted images with voxel based morphometry (VBM) analyses. White matter atrophy appeared in perinatally HIV-infected youths in brain areas including the bilateral posterior corpus callosum (CC), bilateral external capsule, bilateral ventral temporal WM, mid cerebral peduncles, and basal pons over controls. Gray matter volume increase was observed in HIV-infected youths for several regions including the left superior frontal gyrus, inferior occipital gyrus, gyrus rectus, right mid cingulum, parahippocampal gyrus, bilateral inferior temporal gyrus, and middle temporal gyrus compared with controls. Global WM and GM volumes did not differ significantly between groups. These results indicate WM injury in perinatally HIV-infected youths, but the interpretation of the GM results, which appeared as increased regional volumes, is not clear. Further longitudinal studies are needed to clarify if our results represent active ongoing brain infection or toxicity from HIV treatment resulting in neuronal cell swelling and regional increased GM volume. Our findings suggest that assessment of regional GM and WM volume changes, based on VBM procedures, may be an additional measure to assess brain integrity in HIV-infected youths and to evaluate success of current ART therapy for efficacy in the brain.

## Highlights

• First time investigation of gray/white matter changes in HIV-infected youths
• Brain white matter atrophy observed in the HIV-infected youths
• Significantly increased gray matter volume emerged in several regions.
• Pilot findings indicate white matter injury in perinatally HIV-infected youths.

## 1 Introduction

Perinatal infection with HIV remains a major public health problem worldwide disproportionately affecting children in developing countries, with still limited access to antiretroviral therapy (ART). However, in developed countries, the tremendous success of ART has transformed perinatal and adult HIV into a chronic disease with long term survival (Lee et al., 2006; Moore and Chaisson, 1999; Palella et al., 1998). Although brain involvement with HIV is well documented for perinatally infected infants and children (Tardieu et al., 1995), long-term neurologic outcomes for HIV-infected children and youths need further study. Even though newer antiretroviral therapies have decreased the incidence of HIV encephalopathy in perinatally HIV infected children with early aggressive treatment (Patel et al., 2009), many children have survived to adulthood from earlier eras with less efficacious regimens and may experience indolent ongoing brain injury. Recent studies document neurocognitive compromise in the older, treated perinatally infected HIV patients and the need for noninvasive assessment of ongoing brain integrity in long-term treated survivors (Nagarajan et al., 2012; Smith et al., 2012). Improved detection of brain injury could result in treatment modifications to improve the cognitive function of HIV-infected patients.

Both in-vitro and in-vivo studies have demonstrated that tissue changes have been observed in distinct brain regions in patients with HIV. Evidence in post-mortem brain tissue in patients with HIV confirmed wide spread neuronal loss that involved the entire cerebral cortex, basal ganglia and brain-stem structures (Adle-Biassette et al., 1999). Early studies in adults based on brain volumetric MRI reported volume changes in subcortical gray matter structures including the basal ganglia and central white matter (Aylward et al., 1993; Stout et al., 1998) in HIV patients. Subsequent studies, using tensor based morphometry (Chiang et al., 2007) and measurement of cortical gray matter thickness (Thompson et al., 2005), showed severe cortical atrophy and found correlation with cognitive and motor impairment with prefrontal and parietal tissue loss in HIV-infected patients. Furthermore, more recent morphometric studies using deformation based morphometry (Becker et al., 2011) and VBM (Cohen et al., 2010) suggested loss of brain volume in cortical and subcortical regions, despite effective ART. A recent VBM study (Küper et al., 2011) provided evidence for atrophy of nigro-striatal and fronto-striatal circuits in HIV. It showed that the prefrontal gray matter atrophy in HIV was associated with longer disease duration, while motor dysfunction was associated with basal ganglia gray matter atrophy and lower CD4 cell count correlated with occipital gray matter loss.

Although various neuroimaging studies investigated structural brain changes in HIV, regional gray and white matter volume changes have rarely been studied in perinatally HIV-infected youth. Studies in perinatally HIV-infected children using traditional neuroradiographic techniques (computed tomography and MRI) (Johann-Liang et al., 1998) have shown cerebral atrophy and basal ganglia calcification, but these studies were before the widespread use of combination antiretroviral therapy and early diagnosis in infants. In this study, we have compared a group of older perinatally HIV-infected youths with age-matched healthy control group with VBM, using high-resolution T1-weighted images and statistical parametric mapping method. The VBM procedure allows the detection of highly localized differences, consistently observed across the samples, over the whole brain, even in areas where the region of interest analysis would be difficult. The procedure has proved to be a powerful method in detecting regional tissue differences in other cerebral disorders such as Alzheimer's disease, schizophrenia, and amyotrophic lateral sclerosis (ALS) (Ha et al., 2012; Honea et al., 2005; Kassubek et al., 2005), even in clinical conditions where routine imaging does not show any visible abnormality (Padovani et al., 2006; Shin et al., 2006), and provides the opportunity for an unbiased general search of abnormalities in the whole-brain volume (Ashburner and Friston, 2000). Thus VBM may be useful to examine regional gray and white matter changes in perinatally HIV-infected children. The objective of our study was to identify the gray matter (GM) and white matter (WM) changes in children associated with perinatal HIV infection, using high-resolution T1-weighted images with VBM procedures.

## 2 Materials and methods

We investigated 16 patients with HIV infection (mean age ± SD, 17.0 ± 2.9 years; age range, 13–25 years) and 14 age-matched healthy controls (16.3 ± 2.3 years; 13–25 years). Clinical and other characteristics of HIV-infected and control subjects are included in Table 1. Subject population was part of previously-published study (Nagarajan et al., 2012), and included 15 patients infected perinatally from the mother and one patient infected by blood transfusion at less than one year of life. All HIV patients were recruited from Harbor-UCLA Medical Center (Torrance, CA), Miller Children's Hospital of Long Beach (Long Beach, CA), David Geffen School of Medicine at UCLA (Los Angeles, CA) and Children's Hospital Los Angeles (Los Angeles, CA). Fourteen HIV-negative healthy controls were recruited from the general pediatric clinic at Harbor-UCLA Medical Center and also from family members or friends of the patients. Inclusion criteria for HIV-infected subjects were acquisition of HIV in fetal or neonatal period or in first year of life by blood transfusion, current combination ART and right-hand dominance. For control youths, inclusion criteria were confirmation of HIV negative status by buccal scraping (OraSure Technologies, Bethlehem, PA 18015), and right handed status.

Exclusion criteria for all subjects were claustrophobia, pregnancy, current alcohol or other substance use/abuse, current or past attention deficit disorder diagnosis, active depression (based on subject's self description) or other psychiatric diagnosis (based on subject's need for psychiatric medications or treatments), metabolic disturbances, metallic implants, and other brain diseases (not HIV related). For control subjects, exclusion criteria also included any chronic medication other than asthma medication and severe school difficulties. CD4 T cell counts and viral load at time of testing were collected from chart review as was information regarding maternal substance abuse during pregnancy. Informed consent was obtained from all subjects prior to the study, and study protocol was approved by the Institutional Review Boards at both the Harbor-UCLA Medical Center and the University of California at Los Angeles.

### 2.1 Magnetic resonance imaging

All brain imaging studies were performed in a 3.0 Tesla MRI scanner (Siemens, Magnetom, Tim-Trio; Erlangen, Germany), using a 12-channel phased-array head coil. High-resolution T1-weighted images were acquired using a magnetization-prepared-rapid-acquisition-gradient-echo (MP-RAGE) sequence (repetition time = 2200 ms; echo time = 2.34 ms; inversion time = 900 ms; flip angle = 9°; matrix size = 320 × 320; field of view = 230 mm × 230 mm; slice thickness = 0.9 mm; number of slices = 192).

### 2.2 Data processing

Brain imaging data were processed using the statistical parametric mapping package (SPM8, http://www.fil.ion.ucl.ac.uk/spm/), MRIcroN, and MATLAB-based (The MathWorks Inc, Natick, MA) custom software. High-resolution T1-weighted images from all individual subjects were visually-examined for the presence of tumors and cysts. High-resolution T1-weighted images were also examined for any motion artifacts.

High-resolution T1-weighted images corrected for any bias and inhomogeneity-corrected images were partitioned into gray, white, and cerebrospinal fluid (CSF) tissue types using a unified segmentation approach (Ashburner and Friston, 1997; Friston et al., 1995). Gray and white matter tissue maps were normalized to the Montreal Neurological Institute (MNI) space and were modulated and smoothed using a Gaussian filter (full width at half maximum, 10 mm). High-resolution T1-weighted images of all individual subjects were also normalized to MNI space. The normalized images of all subjects were averaged, and averaged images were used as background images for structural identification. Using the segmented gray, white, and CSF tissue probability maps, global GM, WM, and CSF volumes were calculated using an MATLAB based program.

### 2.3 Statistical analysis

We used the Statistical Package for the Social Sciences (SPSS, V 18.0, Chicago, IL) software for assessment of demographic and biophysical data. Numerical data were compared between groups using independent samples t-tests, and categorical data evaluated using the chi-square test. The normalized and smoothed gray and white matter tissue probability maps were compared between groups using analysis of covariance (ANCOVA; uncorrected threshold, p = 0.01; extended threshold, 100 voxels), with age and gender included as covariates. To avoid edge effects around the border between GM and WM, we excluded all voxels with a GM or WM value of < 0.1 and used an explicit mask, derived from mean of GM and WM tissue probability maps. Clusters with significant differences in gray and white matter between groups were overlaid onto background images for structural identification.

## 3 Results

### 3.1 Demographics

There were no significant differences in sex and age between HIV-infected and control groups. Maternal substance abuse was documented for 25% HIV-infected patients, but information regarding maternal substance abuse was not always available.

### 3.2 White matter volume (WMV) changes

**Fig. 1:**
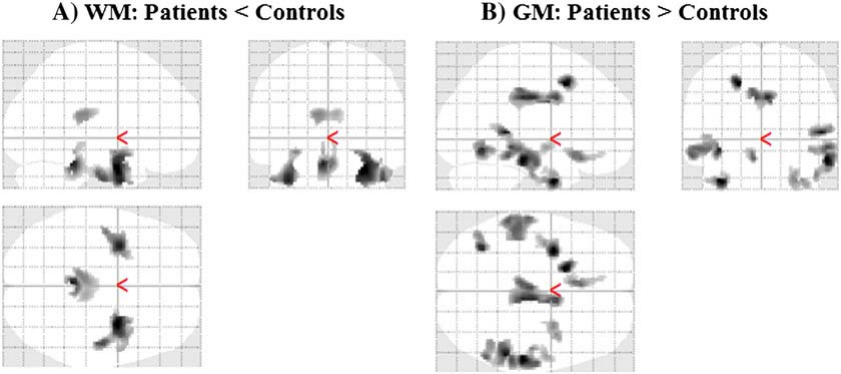
Two-dimensional maximum intensity projection (MIP) glass brain representation showing areas of (A) reduced WM volume in HIV-infected group, compared with controls; (B) elevated GM volume in HIV-infected group, compared with controls.

**Fig. 2:**
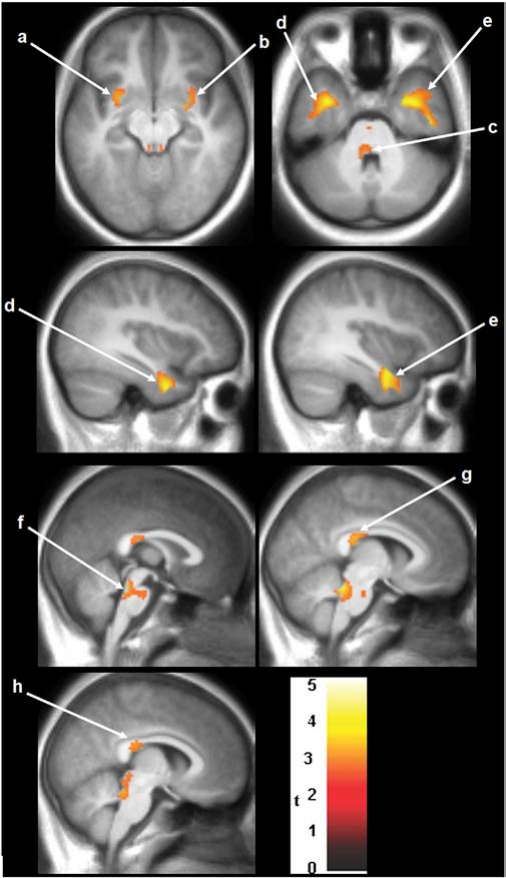
SPM analysis of WMV reduction in patients compared with controls. Results are projected on axial/sagittal/coronal slices of the study specific averaged MPRAGE-image in a standard stereotactic space derived from all the 30 study participants. Bilateral clusters showing lower WM is observed in the external capsule (a, b), ventral temporal WM (d, e), and posterior corpus callosum (g, h). WM damage was also observed in the mid cerebral peduncles (c) and basal pons (f). The neurological convention is adopted, with the left side of the brain on the left side of coronal and axial panels. The extent of variation in damage is provided by the color coded *t*-values. The color scale represents *t*-statistic values, with colored regions exceeding the significance threshold of *P* < 0.01 and minimum cluster of 100 voxels.

White matter volume (WMV) reduction appeared in HIV youths compared with control subjects, and are shown as three views of regions projections to two-dimensional (2D) glass brain panels (Fig. 1A). Reduced WMV appeared in the bilateral posterior corpus callosum (CC), bilateral external capsule, bilateral ventral temporal WM, mid cerebral peduncles, and basal pons (Fig. 2). No regions emerged with a significant elevation of WMV in HIV-infected youths over healthy controls.

### 3.3 Gray matter volume (GMV) changes

**Fig. 3:**
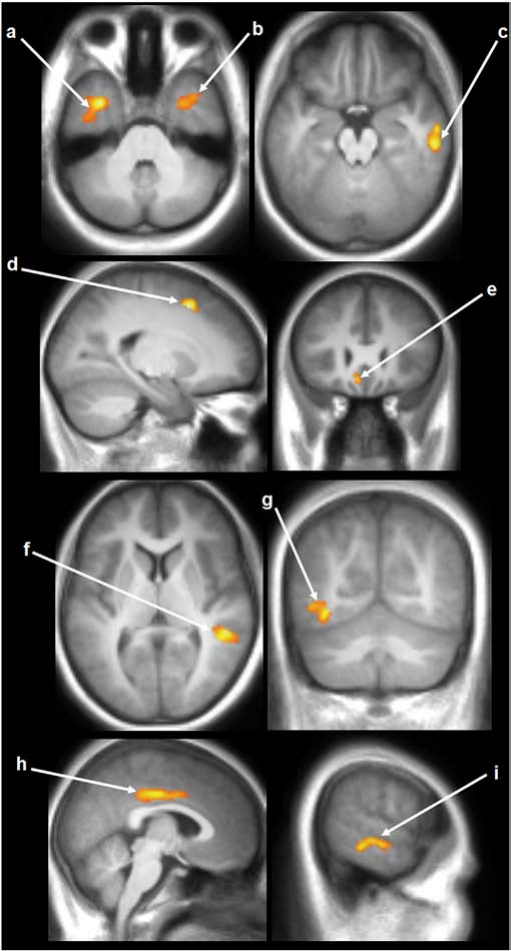
SPM analysis of GMV elevation in patients compared with controls. Results are projected on coronal, sagittal and axial slices of the study specific averaged MPRAGE-image in a standard stereotactic space derived from all the 30 study participants. Bilateral clusters of significantly higher GM values appeared in the inferior temporal gyrus (a, b) and middle temporal gyrus (f, i). Regions of higher GM also appeared in the right parahippocampal gyrus (c), left superior frontal gyrus (d), left gyrus rectus (e), left inferior occipital gyrus (g), and right mid cingulum (h). Conventions as in Fig. 2.

Significantly higher gray matter volumes (GMV) emerged in HIV-infected youths over healthy children, and are displayed in 2D glass brain (Fig. 1B). Higher GMV was observed in the left superior frontal gyrus, inferior occipital gyrus, gyrus rectus, right mid cingulum, parahippocampal gyrus, bilateral inferior temporal gyrus, and middle temporal gyrus in HIV-infected subjects over controls (Fig. 3).

### 3.4 Global volume changes

Global GM and WM of HIV-infected and control subjects are tabulated in Table 1. No significant differences were observed between HIV-infected and healthy control groups for total GM, total WM or brain (GM + WM) to intracranial (GM + WM + CSF) volume ratio, even controlling for age and sex. Mean and standard deviation (SD) GMV for HIV-infected youths was 710.75 cm^3^ (SD = 48.82) and for healthy volunteers was 721.76 cm^3^ (SD = 39.25). Mean and SD of WMV for HIV-infected youths was 475.58 cm^3^ (SD = 48.17) and for healthy volunteers was 494.11 cm^3^ (SD = 53.00).

## 4 Discussion

In this study, we investigated regional GM and WM volume changes in perinatally HIV-infected youths compared with age-matched healthy youths. To our knowledge, the present study is the first characterization of regional brain GM and WM structures using a VBM approach in individuals infected perinatally or early in life with HIV. The major findings in this study are that these HIV-infected individuals receiving ART showed WM atrophy in selected brain regions and significantly higher GMV in other selected regions. We did not observe any significant changes in the global WM and GM volume between the two groups. Also, analysis excluding the one subject with transfusion related HIV did not change the morphometry results.

Several studies on adults have reported reduced white matter volume (Chiang et al., 2007; Stout et al., 1998) in HIV + patients compared with healthy controls. A recent longitudinal study involving region of interest analysis and deformation morphometry showed greater rates of white matter volume loss in HIV + patients on ART than control individuals (Cardenas et al., 2009). Our finding of reduced WMV in the corpus callosum is consistent with a study showing callosal thinning on anatomical MRI (Thompson et al., 2006) and other studies using DTI in HIV-infected adults showing white matter injury in the subcortical white matter, including the frontal lobes, the genu and splenium of the corpus callosum, and the internal capsule (Chang et al., 2008; Filippi et al., 1998; Gongvatana et al., 2009; Pfefferbaum et al., 2007; Pomara et al., 2001; Thurnher et al., 2005; Wu et al., 2006).

Unlike some of the neuronal diseases such as Alzheimer's disease and Parkinson's disease, HIV can also disrupt brain tissue through indirect mechanisms of neural injury mainly as a result of axonal disruption and aberrant sprouting of synaptodendritic connections, often without substantial neuronal loss (Ellis et al., 2007). Synaptodendritic neuronal injury may be an important mediator of cerebral injury in HIV, and our finding of WM volume reduction may be a reflection of these changes. Other considerations include possible inadequate penetration of some antiretroviral medications into the central nervous system with resultant poor control of infection (Cysique et al., 2004). Alternatively, severe damage sustained during brain development may not be ameliorated even by subsequent effective HIV regimens.

In contrast to the WM loss, we identified several areas of elevated GM volume in perinatally-infected HIV + youths compared with healthy controls. Our finding of elevated GM volumes in HIV + patients does not agree with several previous studies in HIV + adults (Becker et al., 2011; Cohen et al., 2010; Thompson et al., 2005). However, one adult study showed GM hypertrophy in the putamen (Castelo et al., 2007), which was not an area of increase identified by our analysis. Similar results of GM volume increase have been observed in other clinical populations including patients with bipolar disorder (Adler et al., 2005), attention deficit hyperactivity disorder (ADHD) (Sowell et al., 2003), and schizophrenia (Dazzan et al., 2005; Deng et al., 2009). An increase in gray matter volume could be due to an increase in cell size, neural or glial cell genesis, or spine volume (Driemeyer et al., 2008; May et al., 2007). Although the etiology of GM volume increases observed in perinatally HIV-infected youths remains unclear since such findings have not been reported in adults with HIV, several issues need to be considered. Since our medical history information regarding maternal substance abuse during pregnancy is incomplete, maternal substance abuse may be a confounding factor. All the HIV + patients were receiving ART medication at the time of the MRI scan and these medications may have a neuroprotective effect and potentially impact neuroanatomic findings during development. Unfortunately, the average age for initiating any antiretroviral therapy was 4.6 years in our subjects and considerable cerebral damage may have already occurred.

Increased GM volume has also been found in children with attention deficit hyperactivity disorder (ADHD) (Sowell et al., 2003) undergoing stimulant drug treatment. Previous or current ADHD diagnosis was an exclusion criterion for our study, so our findings are not related to this confounding diagnosis. There is growing evidence that anti-psychotic drugs used in schizophrenia may result in increased GM volume (Deng et al., 2009), possibly playing a role in mediating neural plasticity during the early phase of clinical recovery. Previous treatment of HIV + patients with either mood stabilizers or antipsychotic medications or current and previous antiretroviral therapy could potentially contribute to cerebral toxicity and the findings of increased GM volumes (Adler et al., 2005).

GM hypertrophy may also be a result of inflammation. It is known that neuropathogenesis of HIV is accompanied by HIV viral protein induced brain inflammation (Kaul et al., 2001; Mattson et al., 2005) and the expression of inflammatory markers are directly involved, which has been supported by in vitro studies (Zhao et al., 2001). Even after the introduction of highly active ART, there is ongoing detectable neuroinflammation (Anthony et al., 2005). Inflammatory activity may be a marker of neurological progression from asymptomatic to symptomatic disease stages in HIV (Chang et al., 2004). Imperfect pruning may also contribute to GMV elevation. Adolescence marks a period of brain development accomplished through synaptic refinement and myelination. During adolescence, synaptic pruning occurs involving reduction in GM due to elimination of unnecessary neural connections (Sowell et al., 2001; Squeglia et al., 2009). It may happen that pruning processes are affected by ART medications or HIV infection, resulting in larger GMV in HIV + youths compared with controls. Potentially either HIV infection directly or ART may result in altered pruning and a larger GMV.

Our previous study using 2D-MRS and neuropsychological tests in this same cohort (Nagarajan et al., 2012) demonstrated metabolic abnormalities as evidenced by elevation of scyllo-inositol (Scy) and glutamate (Glu) ratios with respect to total creatine (tCr) and total choline (tCho) in the HIV-infected youths. However, since this earlier study focused on the right frontal region it cannot be directly related to our whole brain VBM study. We did demonstrate in that earlier study of the same population that neurocognitive function was impaired in the HIV-infected group as evidenced by lower scores for the attention/processing speed domain, but no correlation was found between metabolites and neurocognitive performance. In the present study we have not also found any correlation between the structural changes and the neurocognitive performance.

Major limitations of this study are the small sample size and the cross sectional design. Future longitudinal studies on perinatally HIV-infected subjects are needed to confirm our results and should be conducted using a larger study population with neurocognitive performance correlation. Other imaging modalities, including high angular resolution diffusion imaging, may be another way to investigate regional WM changes.

## 5 Conclusions

In this study we found white matter atrophy in perinatally HIV-infected youths in the bilateral posterior CC (in agreement with adult studies) and other brain areas including external capsule, ventral temporal WM, mid cerebral peduncles, and basal pons compared with controls. Increased gray matter volume emerged in several regions including the left superior frontal gyrus, inferior occipital gyrus, gyrus rectus, right mid cingulum, parahippocampal gyrus, bilateral inferior temporal gyrus, and middle temporal gyrus. These results provide evidence of changes in both the GM and WM, which may indicate early developmental cerebral injury in perinatally infected HIV + youths, ongoing HIV infection in the brain despite ART, or toxicity of HIV medications. Our findings suggest that HIV + patients on successful ART should be observed long term for both direct and ongoing effects of the virus on the brain and possible toxic effects of antiretroviral medications. The most neuroprotective regimens must be developed for both perinatally-infected patients and patients infected as adults and improved noninvasive sequential imaging of the brain may assist in elucidating the best treatments.

## Figures and Tables

**Table 1:** Characteristics of study participants.

	HIV + patients	Healthy controls	p-Value
*N*	16	14	N/A
Age	17.0 ± 2.9 years	16.3 ± 2.3 years	0.48
Gender (male/female)	8/8	9/5	0.45
CD4 count	536 ± 340	N/A	N/A
%CD4 > 200	81.2 (n = 13)	N/A	N/A
Log viral load	4.7 (1.3)	N/A	N/A
% Log viral load < 1.9	56 (n = 9)	N/A	N/A
Age at first HIV treatment	4.6 ± 4.8 years	N/A	N/A
% treated at less than one year	25 (n = 4)	N/A	N/A
Total gray matter (cm^3^)	710.75 ± 48.82	721.762 ± 39.25	0.50
Total white matter (cm^3^)	475.58 ± 48.17	494.11 ± 53.00	0.33
Brain/intracranial ratio	0.86 ± 0.01	0.87 ± 0.01	0.85

N/A = Not applicable or available.
